# Determinants of Persistent Patterns of Pepino Mosaic Virus Mixed Infections

**DOI:** 10.3389/fmicb.2021.694492

**Published:** 2021-07-06

**Authors:** Cristina Alcaide, Miguel A. Aranda

**Affiliations:** Department of Stress Biology and Plant Pathology, Centro de Edafología y Biología Aplicada del Segura (CEBAS)-CSIC, Murcia, Spain

**Keywords:** *potexvirus*, super-infection exclusion, tomato, virus transmission, viral diversity

## Abstract

Pepino mosaic virus (PepMV) has become a pandemic virus in tomato crops, causing important economic losses worldwide. In Spain, isolates of the EU and CH2 strains co-circulate, with PepMV-EU predominantly found in mixed infections. Simultaneous *in planta* mixed infections result in an asymmetric antagonism against PepMV-CH2, but the outcome of over-infections has never been tested. PepMV-EU and PepMV-CH2 time-lagged inoculations were performed, and viral accumulation was measured 10 days after challenge inoculation. PepMV-EU had a protective effect over PepMV-CH2; in contrast, the accumulation of PepMV-EU increased in plants pre-inoculated with PepMV-CH2 as compared to single infections. We also studied the effect of the type of infection on viral transmission. Independently of the nature of the infection (single or mixed), we observed a strong positive correlation between virus accumulation in the source plant and transmission, excluding mixed infection effects different than modulating viral accumulation. Finally, in order to determine the genetic variability of PepMV strains in single and mixed infections, a 430 nucleotide region was RT-PCR amplified from samples from a serial passages experiment and deep-sequenced. No significant differences were found in the number of nucleotide substitutions between single and mixed infections for PepMV-EU; in contrast, significant differences were found for PepMV-CH2, which was more variable in single than in mixed infections. Comparing PepMV-EU with PepMV-CH2, a higher nucleotide diversity was found for PepMV-CH2. Collectively, our data strongly suggest that PepMV mixed infections can impact the virus epidemiology by modulating *in planta* virus strain accumulation and diversification.

## Introduction

Pepino mosaic virus (PepMV) (family *Alphaflexiviridae*, genus *Potexvirus*) is a positive-sense single-stranded RNA virus which affects tomato crops worldwide (Hanssen and Thomma, 2010; Gómez et al., 2012). The PepMV genome is approximately 6.4 kb in length and contains five open reading frames (ORFs) flanked by two untranslated regions and a polyadenylated tail at the 3′ end (Aguilar et al., 2002). ORF1 encodes an RNA-dependent RNA polymerase (RdRp) which encompasses three conserved domains, a methyltransferase, an NTP-binding site, and the polymerase domains. ORFs 2, 3, and 4 form the triple gene block (TGB), encoding proteins TGB1, TGB2, and TGB3, which are involved in the viral intra- and inter-cellular movement and the building of the viral replication complexes (Morozov and Solovyev, 2003). TGB1 is a protein with both RNA binding and RNA silencing suppression activities (Morozov and Solovyev, 2003; Solovyev et al., 2012; Mathioudakis et al., 2014), while TGB2 and TGB3 target cellular endomembranes and are capable of directing TGB1 to plasmodesmata (Tilsner et al., 2013). ORF5 encodes the capsid protein (CP), which apart from its structural role (Agirrezabala et al., 2015), is involved in viral cell to cell and long distance movements (Morozov and Solovyev, 2003; Sempere et al., 2011), and is also a suppressor of RNA silencing, at least for PepMV (Mathioudakis et al., 2014).

PepMV isolates can be classified into five strains: the European (EU), the Chilean (CH2), the North American (US1/CH1), the original Peruvian (LP), and the new Peruvian (PES) strains. Also a US recombinant isolate (US2) has been described (Hanssen and Thomma, 2010; Moreno-Perez et al., 2014). In Spain, PepMV populations infecting tomato crops were composed of isolates belonging to the EU strain until 2005, when viral populations underwent a shift to become mainly formed by isolates belonging to the CH2 strain; isolates of the EU strain were maintained in minor proportions in mixed infections (Pagán et al., 2006; Gómez et al., 2009). This epidemiological scenario seems to be long-lasting (Gómez-Aix et al., 2019a; Alcaide et al., 2020a), in spite of the superior *in planta* fitness of CH2 isolates as compared to EU isolates (Gómez et al., 2009; Alcaide et al., 2020a). Broadening our understanding of this phenomenon is highly important, as the maintenance of mixed infections may have critical consequences on the PepMV evolutionary outcome.

Mixed viral infections are common in plants. Mixed infections can lead to a variety of viral interactions, which may affect both the fitness and the genetic diversity of the viruses involved (Syller, 2012; Alcaide et al., 2020b). It has been described that at the beginning of the infection of a host, multiple viral infections within a cell increase the chances of disadvantageous or neutral mutants to arise and be maintained by complementation. These mutants are less likely to be fixed in subsequent stages of the infection, where genetic drift can randomly fix the most abundant variants, and negative selection can filter disadvantageous mutations, leading to the fixation of the genotypes that confer superior fitness (Wodarz et al., 2019). Viral fitness can vary in mixed infections compared to single infections, which can also affect viral transmission rate, virus virulence, host range, and symptomatology (Alcaide et al., 2020b; Moreno and López-Moya, 2020). Mixed infections in plants can occur with related or unrelated viruses. For related viruses, exclusion of super-infection can occur; this is a frequently-described phenomenon consisting of the exclusion of an incoming virus in a host that has been previously infected with another related virus (Folimonova et al., 2010; Ziebell and Carr, 2010). A variant of this phenomenon is called cross-protection, in which infection of a plant with a mild strain of a virus prevents or delays infection with a severe strain (Hull, 2014). Cross-protection was first described almost a century ago (Mckinney, 1929), and has since been exploited as a virus control strategy for several virus/host combinations (Ziebell and Carr, 2010), including PepMV (e.g., Agüero et al., 2018).

The epidemiology of PepMV in Spain has been extensively studied. However, there is still insufficient knowledge about the factors that influence the maintenance of PepMV strain mixed infections in nature and their evolutionary consequences. In contrast with data from single infections, CH2 isolates have been shown to have a reduced *in planta* fitness when they were simultaneously inoculated with an EU isolate, while simultaneous mixed infections had no impact on the fitness of the EU isolate (Gómez et al., 2009; Alcaide et al., 2020a). By studying the PepMV genetic diversity in two tomato producing areas with differing patterns of single and mixed infections predominance, we showed that the CH2 population was more diverse in the area where both PepMV strains had been mixed-infecting plants for years. This was suggestive of differential evolutionary forces operating over the CH2 populations under single or mixed infections (Alcaide et al., 2020a). In the same study, we also showed that the *in planta* fitness of CH2 isolates from both geographical locations was similar, suggesting that the observed variability had a neutral adaptive value (Alcaide et al., 2020a), ruling out a primary effect of mixed infections on selection. Lastly, in an earlier study, we showed that CH2 and EU simultaneous mixed infections resulted in symptom amelioration in tomato plants and host range broadening (Gómez et al., 2009), leading us to hypothesize that these factors could be responsible for the maintenance of PepMV mixed infections, although other ecological factors may also play a role. The work that we describe here was conducted to identify additional determinants of persistent patterns of PepMV mixed infections, including the outcome of over-infections with isolates of the heterologous strains, the influence of mixed infections in virus transmission, and the incidence of mixed infections on virus diversification.

## Materials and Methods

### Plant Material, Plant Growth, Virus Isolates, and Virus Inoculation

Tomato (cv. Moneymaker) seeds were germinated in Petri dishes at 28°C in darkness for 3 days and transplanted to substrate (coconut fiber, peat, and perlite in a 5:10:1 proportion) in 1-L plastic pots. Plants were grown in a controlled environment greenhouse with conditions set at 16/8 h photoperiod and 26/22°C in a day/night cycle. The virus isolates used for inoculations were PepMV-Sp13 (Aguilar et al., 2002) and PepMV-PS5 (Gómez et al., 2009), prototypes of the EU and CH2 strains, respectively; we will refer to them as PepMV-EU (equivalent to PepMV-Sp13 in this paper) and PepMV-CH2 (equivalent to PepMV-PS5). Inoculations were performed using virion preparations at 100 μg/mL in phosphate buffer (K_2_HPO_4_ + KH_2_PO_4_) pH 8.0 as described before (Gómez et al., 2009) unless otherwise stated. For simultaneous infections, virions were mixed in a proportion 1:1 in equal concentrations.

For the experiment conducted to test the effect of the age of the plant on the outcome of simultaneous mixed inoculations, three tomato plants were inoculated with PepMV in single or mixed infections (PepMV-EU + PepMV-CH2) at different time points, 28, 31, 33, 35, and 39 days post germination, in the second, third, fourth, fourth again, and fifth true leaves, respectively. Then, 10 days post infection samples were collected and stored at −80°C before further analyses (see below). For the experiments conducted to test virus accumulation after over-inoculations, 15 tomato plants were mechanically inoculated with PepMV-EU or PepMV-CH2. Afterward, time-lagged inoculations with the heterologous virus (challenge virus) were performed in three plants at 3, 6, 8, 10, or 14 days after the first inoculation. Additionally, three tomato plants were co-inoculated with both viruses at the same time (time 0) and another three plants were single inoculated with PepMV-EU or PepMV-CH2. Ten days after the second inoculation (or the first inoculation in the case of single and simultaneous infections), the leaves that emerged after the last inoculated leaf were collected and stored at −80°C before further analyses (see below). This experiment was repeated twice. For the experiment on the efficiency of PepMV mechanical transmission, five plants from the experiment described above from treatments with different types of infections (single, simultaneous, and over-infections) were used as inoculum sources. Extracts used for inoculations were prepared by grinding 250 mg of plant tissue in 1 mL of phosphate buffer pH 8; virus concentration (see below) was measured in these extracts. Then, two dilutions were performed in phosphate buffer. Fifty-four tomato plants were inoculated for each treatment; 18 plants with the undiluted extract, and another 18 plants with each of the 1:5 and 1:25 dilutions in phosphate buffer. Ten days after inoculation, a tissue print (see below) for each of the plants was performed. To study the effects of mixed infections on PepMV diversification, we took advantage of the serial passages experiment performed by Agüero et al. (2018).

### RNA Extractions and Viral Quantification

Plant samples were ground in liquid nitrogen, and RNA was extracted using the NZY Total RNA Isolation kit (NZYTech, Portugal) following the manufacturer’s instructions. Extracts were treated with DNaseI (Sigma-Aldrich), and viral accumulation was quantified by RT-qPCR using the One-step NZYSpeedy RT-qPCR Green Kit and ROX Plus (NZYTech, Portugal) with specific primers for PepMV-EU and PepMV-CH2 (Gómez et al., 2009). To prepare viral RNA as pure as possible, virion purification was performed as in Agirrezabala et al. (2015), and RNA extraction from virions was performed following the protocol described by AbouHaidar et al. (1998). Then, 10-fold serial dilutions of the viral RNA from disassembled PepMV-EU or PepMV-CH2 virions were used to prepare the standard curves used for viral quantification.

### Tissue-Printing Hybridization

For virus detection in tissue prints, two leaf petioles from each plant were sectioned with a fresh razor blade and blotted onto positively-charged nylon membranes (Amersham Hybond-N^+^, GE Healthcare, United Kingdom). For membrane pre-hybridization, hybridization, washes, and luminescence detection, we followed the protocol described by Marco et al. (2003). RNA probes specific for each virus strain were used (Gómez-Aix et al., 2019b). To detect luminescence, an Amersham^TM^ Imager 600 chemiluminescent detector was used.

### Amplification and Deep Sequencing of a Region of the PepMV Genome

The plant material stored frozen at −80°C from the evolution experiment described in Agüero et al. (2018) was used. Specifically, we used material from passages 1, 2, 4, 6, and 8, corresponding to the treatments of single and mixed infections of PepMV-EU and PepMV-CH2. After RNA extraction, a strain-specific reverse transcription was carried out using Reverse Transcriptase (Roche, Germany) and the primers described by Alcaide et al. (2021). A sequence of 415 nucleotides for PepMV-EU and 421 pairs for PepMV-CH2, corresponding to a region of the viral replicase, was PCR-amplified by using Phusion DNA polymerase (New England Biolabs, Canada) and strain-specific primers with Illumina adaptors (5′ TCGTCGGCAGCGTCAGATGTGTATAAGAGACAGttcctatac tattaagactg 3′ and 5′ GTCTCGTGGGCTCGGAGATGTGTAT AAGAGACAGgtgtggcaatgctggg 3′ for PepMV-EU; 5′ TCGT CGGCAGCGTCAGATGTGTATAAGAGACAGggacttctcttacacc 3′ and 5′ GTCTCGTGGGCTCGGAGATGTGTATAAGAGAC AGttaacatgtgcagggggcaa 3′ for PepMV-CH2). Upper letters correspond to Illumina adaptors sequences and lower letters correspond to viral sequences. We targeted this region as it is the more dissimilar region in the PepMV genome when comparing the EU and CH2 strains (Gómez-Aix et al., 2019b) and the most variable based on our experience (Alcaide et al., 2021). Amplicons were sequenced in a lane of a MiSeq platform (Macrogen Inc., South Korea), yielding 300 bp paired-end reads.

### HTS Data Analysis

FASTQC was used to determine the quality of the raw data (Andrews, 2014), and Trimmomatic (Bolger et al., 2014) to trim low quality bases. Reads were mapped against the reference genomes using bwa (Li and Durbin, 2009), and alignments were processed with SAMtools (Li et al., 2009). SNP calling was performed with LoFreq (Wilm et al., 2012), and the true variants considered were those with a frequency >0.01. Finally, the SNPGenie software (Nelson et al., 2015) was used to estimate the nucleotide diversity, π, defined as the mean number of pairwise differences per site in the pooled sample across the sequenced region.

### Statistical Analysis

Statistical analyses were carried out using IBM SPSS Statistics v26. General linear models were used to determine differences between treatments. The Spearman correlation analyses were performed in R, where the S statistic is the sum of all squared rank differences and ρ the Spearman correlation coefficient.

### Data Availability

Data from HTS have been deposited in the NCBI GenBank with the BioProject code PRJNA718740, under accession numbers SAMN18559886–SAMN18559945.

## Results

### Outcome of Over-Infections With Heterologous Isolates

**FIGURE 1:**
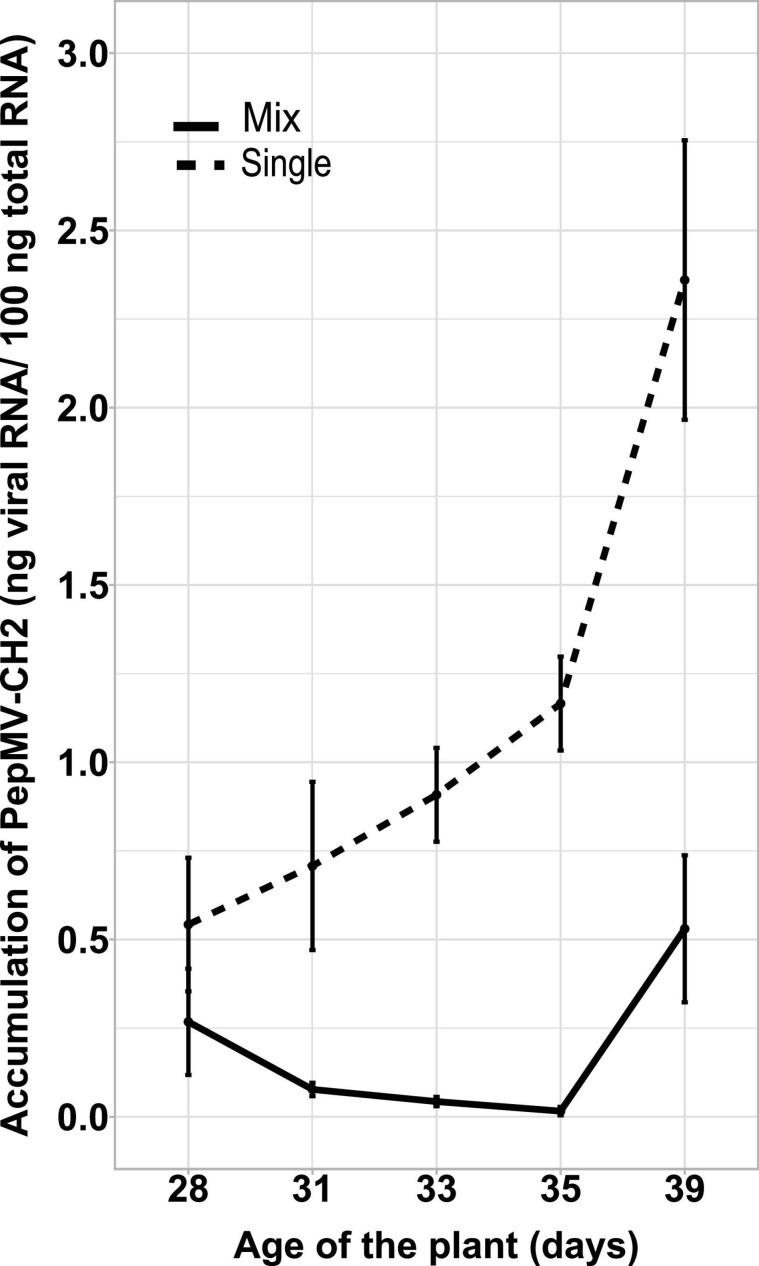
Effect of the plants’ age on the PepMV-EU antagonism over PepMV-CH2 in mixed infections. PepMV-CH2 accumulation was measured in single and mixed PepMV-CH2 + PepMV-EU infections in plants inoculated at different ages. Accumulation was measured by absolute RT-qPCR and shown as ng viral RNA/100 ng total RNA from three plants at 28, 31, 33, 35, and 39 days post germination in single (continuous line) and mixed infected (dashed line) plants.

**FIGURE 2:**
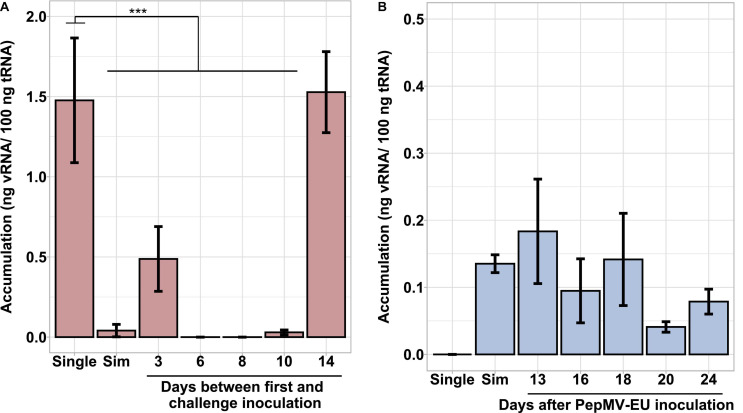
Effect of PepMV-CH2 over-infection on virus accumulation. PepMV-CH2 **(A)** and PepMV-EU **(B)** accumulation was measured in plants pre-inoculated with PepMV-EU and challenged with PepMV-CH2. Single PepMV-CH2 and simultaneous infections with PepMV-EU + PepMV-CH2 were included as controls. Over-inoculations were carried out at different times after pre-inoculation (3, 6, 8, 10, and 14 days). Viral accumulation was determined for three plants for each time point by absolute RT-qPCR and shown as ng viral RNA/100 ng total RNA; each bar represents the mean and its standard deviation. Asterisks show significance level (***p < 0.001; ns, no significant).

**FIGURE 3:**
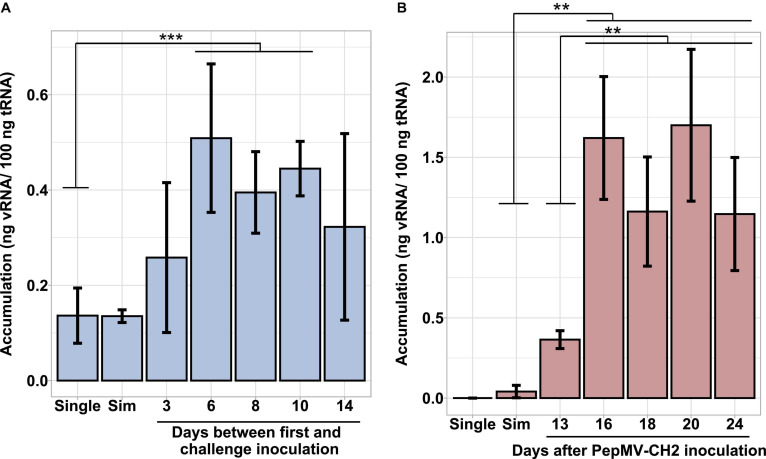
Effect of PepMV-EU over-infection on virus accumulation. PepMV-EU **(A)** and PepMV-CH2 **(B)** accumulation was measured in plants pre-inoculated with PepMV-CH2 and challenged with PepMV-EU. Single PepMV-EU and simultaneous infections between PepMV-EU + PepMV-CH2 were included as controls. Over-inoculations were carried out at different times after pre-inoculation (3, 6, 8, 10, and 14 days). Viral accumulation was determined for three plants for each time point by absolute RT-qPCR and showed as ng viral RNA/100 ng total RNA; each bar represents the mean and its standard deviation. Asterisks show significance level (**p < 0.01; ***p < 0.001).

Simultaneous PepMV-CH2 and PepMV-EU mixed infections did not affect the accumulation of PepMV-EU, but resulted in the decreased accumulation of PepMV-CH2 as compared to single infections. This had already been tested by inoculating young tomato plants and measuring virus accumulation throughout time course experiments, the trend was robust, and the results were shared by all time points in all experiments reported (Gómez et al., 2009; Agüero et al., 2018; Alcaide et al., 2020a). However, the potential effect of the age of the plants was never tested. We first set out to test this variable by single and mixed-inoculating young tomato plants at 2–4 days intervals during a 14-day period. All seeds were sown and plants transplanted at the same time, so the plants were inoculated at different ages. After measuring the accumulation of PepMV-CH2, a steady increase on viral RNA content relative to cellular RNA content could be observed in single infections. Accumulation of PepMV-CH2 was always higher in single than in double infections (Figure 1), ruling out a qualitative effect of the plant age on the PepMV-EU *vs* PepMV-CH2 antagonism at least during the temporal window considered. We next sought out to analyze the effect of delayed over-infections on virus accumulation. Due to super infection exclusion, the outcome of mixed infections may be different depending on whether they occurred simultaneously or delayed. Tomato plants were pre-inoculated with either PepMV-EU or PepMV-CH2 and, at different times after the first inoculation, they were challenge-inoculated with the heterologous virus isolate. The accumulation of each virus was measured in all cases at 10 days post-challenge-inoculation, as in the previous experiment, and similar temporal windows were used. When plants were pre-inoculated with PepMV-EU and challenged with PepMV-CH2 (Figure 2), the accumulation of the latter appeared to be repressed as compared to single infections, similarly to what was observed after simultaneous mixed infection. More specifically, significant differences were found on PepMV-CH2 accumulation when challenge-inoculation took place at 3 (*F* = 15.29, *p* = 0.017), 6 (*F* = 43.26, *p* = 0.003), 8 (*F* = 43.26, *p* = 0.003), and 10 (*F* = 41.48, *p* = 0.003) days after the PepMV-EU inoculation as compared to single infection (Figure 2A). In the same plants, the accumulation of PepMV-EU did not change significantly after challenge inoculation (Figure 2B). These results suggest that the antagonistic interaction of PepMV-EU with PepMV-CH2 was maintained after PepMV-CH2 over-infections, at least when PepMV-CH2 was inoculated after short periods of time (until 10 days) after PepMV-EU inoculation. When plants were pre-inoculated with PepMV-CH2 and challenged with PepMV-EU (Figure 3), the accumulation of the latter appeared to be stimulated as compared to single infections, and this was at odds with what occurred after simultaneous mixed infections; in particular, significant differences were found on the accumulation of PepMV-EU when it was challenge-inoculated at 6 (*F* = 15.06, *p* = 0.018), 8 (*F* = 18.77, *p* = 0.012), and 10 (*F* = 43.11, *p* = 0.003) days after the PepMV-CH2 inoculation as compared to single infections (Figure 3A). We also measured the accumulation of PepMV-CH2 in plants challenged with PepMV-EU. In this case, the challenge inoculation did not seem to result in repression of PepMV-CH2 accumulation, at least at later times after the challenge, as the accumulation of this virus was significantly higher at 6, 8, 10, and 14 days for challenge inoculation (16, 18, 20, and 24 days after PepMV-CH2 inoculation, respectively) compared to simultaneous (*F* = 11.61, *p* = 0.004) or 3 (*F* = 16.94, *p* = 0.001) days for challenge inoculation (13 days after PepMV-CH2 inoculation) (Figure 3B). The over-infection results were robust and reproducible, as illustrated by data from an independent repetition of the experiment in a different time of the year (Supplementary Figure 1). Overall, our results indicate a synergistic interaction that was favorable to PepMV-EU when PepMV-CH2 was inoculated first, and an antagonistic interaction against PepMV-CH2 in mixed infections with PepMV-EU.

### Effect of Mixed Infections on Virus Transmission

**FIGURE 4:**
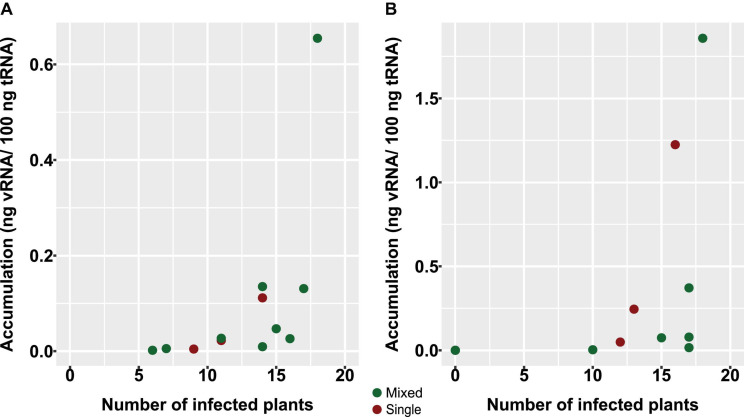
Effect on PepMV mechanical transmission of virus accumulation in source plants. Scatter plots of viral accumulation in source plants, measured by absolute RT-qPCR and shown as ng viral RNA/100 ng total RNA, and efficiency of transmission, measured by the number of infected plants with PepMV-EU **(A)** or PepMV-CH2 **(B)**. Five tomato plants were used as the source of inoculum, including two single infections, one simultaneous mixed infection, and two over infections. Viral load in inocula ranged from 0.05 to 0.66 ng vRNA/100 ng total RNA for PepMV-EU and 2.75E-5 to 1.86 ng vRNA/100 ng total RNA for PepMV-CH2. Undiluted and two dilutions (1:5 and 1:25) of inocula were used to mechanically inoculate 54 plants per treatment. The type of infection was determined for each plant by specific tissue-print hybridization at 10 days after inoculation. Inocula from single and mixed infections (simultaneous or over-infections) are marked in red or green, respectively.

Although other means of transmission have been reported for PepMV, the short-distance transmission of this virus within tomato crops occurs mainly mechanically through the frequent hands-on activities needed for intensive tomato cultivation (Gómez et al., 2012). With the experiment described next, we sought to analyze the effect of mixed infections on PepMV mechanical transmission. Five tomato plants from the experiment above were used as the source of inoculum, including two single infections, one simultaneous mixed infection, and two over-infections. Extracts were prepared from these plants, and viral load was measured by RT-qPCR, providing values ranging from 0.05 to 0.66 ng vRNA/100 ng total RNA for PepMV-EU and 2.75E-5 to 1.86 ng vRNA/100 ng total RNA for PepMV-CH2. Undiluted and two dilutions (1:5 and 1:25) of the extracts were used to mechanically inoculate 54 plants per treatment. After 10 days, the virus strains infecting each plant were determined by specific tissue-print hybridization (Table 1 and Supplementary Table 1). For PepMV-EU, simultaneous mixed infections in the inoculum source resulted in transmission percentages that were similar to a single infection [59.3% (55.6 + 3.7%) *vs* 63.0%], whereas PepMV-EU over-infections in the inoculum source resulted in increased transmission of this virus as compared to single infection [94.4% (3.7 + 90.7%) *vs* 63.0%]. For PepMV-CH2, simultaneous mixed infections in the inoculum source again gave rise to transmission percentages similar to single infection [81.5% (25.9 + 55.6%) *vs* 74.1%], whereas PepMV-CH2 over-infections in the inoculum source resulted in suppressed transmission of this virus as compared to single infections (0 *vs* 74.1%) (Table 1). These data suggested that no bias existed toward mixed or single infections when using mixed-infected plants as the inoculum source, except for the effect of the virus titer in the source plant. Thus, we plotted transmission *vs* virus accumulation in the source plant (Figure 4), and a Spearman correlation analysis was performed to verify if these two variables were associated. We determined a strong positive correlation between virus accumulation and transmission, either considering both viruses at the same time (*S* = 441.09, *p*-value < 0.001, ρ = 0.81; statistical power = 0.99), or separating the data for PepMV-EU (*S* = 55.98, *p*-value = 0.002, ρ = 0.80; statistical power = 0.93) or PepMV-CH2 (*S* = 50.69, *p*-value = 0.001, ρ = 0.82; statistical power = 0.95) (Figure 4). Taken together, our results strongly suggest an important impact of mixed infections on virus transmission through their effects on virus accumulation in the plant source of inoculum.

**TABLE 1:** Efficiency of PepMV-EU and -CH2 mechanical transmission.

**Source plant^1^**	**None^2^**	**EU^3^**	**CH2^3^**	**EU + CH2^3^**
Single EU	20(37.0%)	34(63.0%)	0(0%)	0(0%)
Single CH2	14(25.9%)	0(0%)	40(74.1%)	0(0%)
Simultaneous EU + CH2	8(14.8%)	2(3.7%)	14(25.9%)	30(55.6%)
Over-infection CH2	19(35.2%)	35(64.8%)	0(0%)	0(0%)
Over-infection EU	2(3.7%)	2(3.7%)	1(1.9%)	49(90.7%)

*^1^Five tomato plants were used as source of inoculum, including two single infections, one simultaneous mixed infection, and two over-infections. Viral load in inocula ranged from 0.05 to 0.66 ng vRNA/100 ng total RNA for PepMV-EU, and 2.75E-5 to 1.86 ng vRNA/100 ng total RNA for PepMV-CH2. Eighteen plants were mechanically inoculated with undiluted or a dilution (1:5 or 1:25) of inocula (54 plants per treatment). The type of infection was determined for each plant by specific tissue-print hybridization 10 days after inoculation.*

*^2^The number and percentage of non-infected plants are shown.*

*^3^Each column shows the number and the percentage of plants infected with each PepMV isolate (EU, CH2, or both) for each treatment.*

### Effect of Mixed Infections on PepMV Genetic Diversification

**FIGURE 5:**
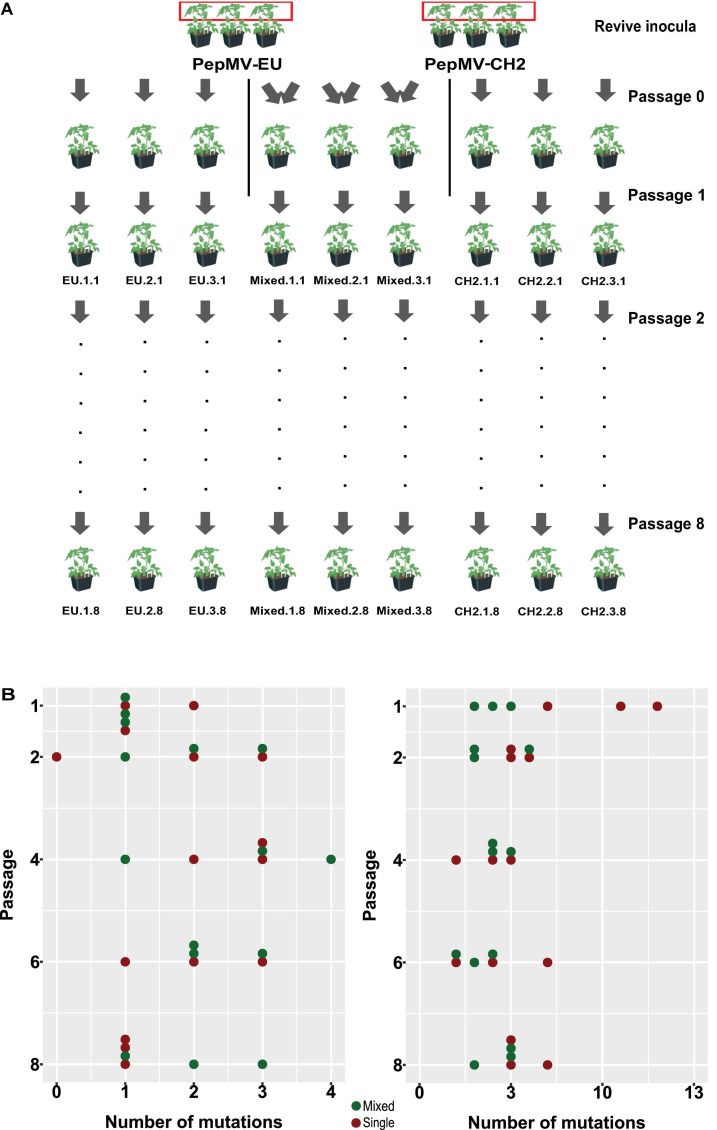
Effect of mixed infections on PepMV genetic diversification. **(A)** Diagram modified from Agüero et al. (2018) illustrating the setting up of founder plants and the passaging of PepMV lineages. Viral populations were passaged every 10–12 days to healthy tomato plants (cv. Moneymaker). A region of 420 nucleotides from the PepMV RdRp was RT-PCR amplified and deep-sequenced for the three independent lineages of PepMV-EU, PepMV-CH2, and PepMV-EU + CH2 from passages 1, 2, 4, 6, and 8. **(B)** Scatter plots between the number of passage (1, 2, 4, 6, and 8) and number of mutations found in the PepMV-EU (top) and PepMV-CH2 (bottom) genomes. Each point represents a lineage; lineages from single or mixed infected plants are marked in red or green, respectively.

**FIGURE 6:**
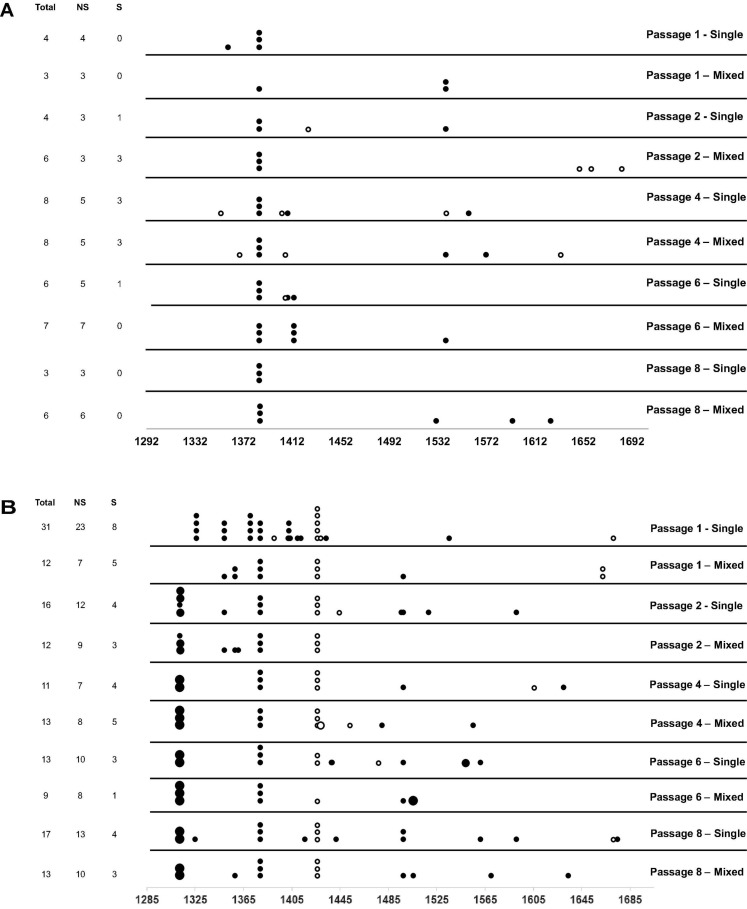
Number and position of mutations identified for the PepMV-EU **(A)** and PepMV-CH2 **(B)** genomic regions sequenced at each passage for single and mixed infections (see diagram in Figure 5A). On the left, the number of mutations, total, non-synonymous (NS), and synonymous (S). Each point represents a mutation and its size represents its frequency; in black, non-synonymous (NS) and in white synonymous (S) substitutions.

**FIGURE 7:**
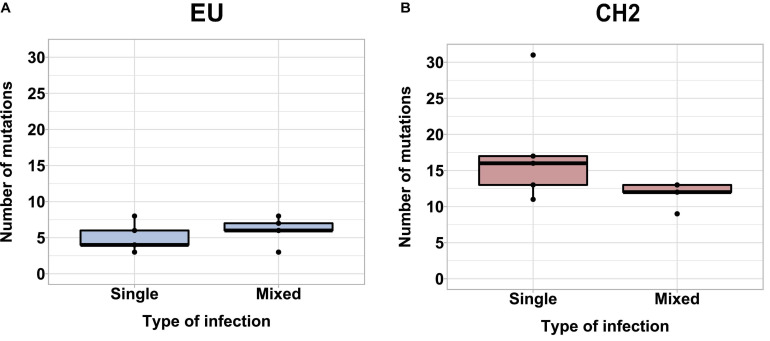
Boxplot showing the average number of mutations for PepMV-EU **(A)** and PepMV-CH2 **(B)** in single and mixed infections pooling together sequences from all the passages of the evolution experiment (see the diagram in Figure 5A).

We next hypothesized that mixed infections could also have an impact on PepMV genetic diversification through the modulation of virus accumulation; specifically, the reduction of PepMV-CH2 accumulation in mixed infections could cause genetic drift associated to bottlenecks during transmission (Acosta-Leal et al., 2011; Kutnjak et al., 2017). To test this hypothesis, we took advantage of the passaging experiment conducted by Agüero et al. (2018), and deep-sequenced a stretch of 420 nucleotides within the PepMV RdRp coding sequence (Gómez-Aix et al., 2019b). We prepared cDNA amplicons using RNA extracts from individual plants after passages 1, 2, 4, 6, and 8 from the three independent PepMV-EU, PepMV-CH2, and PepMV-EU + CH2 lineages (Figure 5A). We deep-sequenced the amplicons and compared the sequences against those of the infectious clones used to initiate these lineages, assuming that these corresponded to the ancestral sequences. After cleaning and trimming the raw NGS data, we obtained between 301,708 and 460,475 reads per sample, resulting in sequencing depths ranging from 207,026 X to 308,183 X. We identified SNPs and evaluated their frequencies in the samples (Supplementary Figure 2), excluding the SNPs with a frequency <0.01. We then plotted the number of mutations for each passage (Figure 5B). No correlations were found between the passage number and the number of mutations, neither for PepMV-EU (*S* = 3817, *p*-value = 0.426, ρ = 0.15) nor for PepMV-CH2 (*S* = 5320, *p*-value = 0.331, ρ = −0.18). We also estimated the nucleotide diversity (π) for either lineage and passage, or pooling together the sequences from the three lineages for each passage. This genetic diversity estimator was found to be of a similar order of magnitude for the independent lineages and for the pooled sequences for each passage (Tables 2, 3), suggesting that there was no differentiation among lineages. We next plotted mutations along each virus sequence for single and mixed infections (Figure 6). For PepMV-EU, the mutations identified ranged in frequency from 0.010 to 0.106. Nucleotide positions 1,386 and 1,532 appeared to be more variable, but mutations in these positions still represented a minority and never became fixed (Figure 6A). For PepMV-CH2, the mutations identified ranged from 0.010 to 0.998 in frequency. There were three obvious variability hotspots at positions 1,312, 1,379, and 1,426 (Figure 6B). The mutated sequences were a minority for positions 1,379 and 1,426, while mutation T1312(G/A), which appeared in passage 2, soon became predominant, although revertant populations were identified afterward for some passages in both, single, and mixed infections (Table 4). For PepMV-CH2, significant differences were found in the number of mutations between single and mixed infections (*F* = 8.00, *p* = 0.010) and among passages (*F* = 3.79, *p* = 0.019), with more mutations in single infections. By comparing the number of SNPs for each virus either in single or mixed infections (Figure 7), significant differences (*F* = 63.41, *p* < 0.001) were found between PepMV-CH2 and PepMV-EU, with a greater number of mutations and higher nucleotide diversity for PepMV-CH2. Taken the above data together, it appears that under the experimental conditions used by Agüero et al. (2018), the swarm of *in planta* variants was larger for PepMV-CH2 than for PepMV-EU, although it remained rather constant with passaging, with mixed infections not associating with evident genetic bottlenecks.

**TABLE 2:** PepMV-EU nucleotide diversity (π) along passages.

	**Single infection**				**Mixed infection**			
	**L1^1^**	**L2**	**L3**	**L1 + L2 + L3^2^**	**L1**	**L2**	**L3**	**L1 + L2 + L3**
P1^3^	8.72E-05	1.79E-04	9.06E-05	8.78E-05	9.37E-05	6.70E-05	1.65E-04	1.42E-04
P2	3.83E-04	2.46E-04	8.99E-05	2.28E-04	1.75E-04	8.84E-05	2.29E-04	8.77E-05
P4	1.74E-04	2.69E-04	5.28E-04	1.73E-04	7.45E-04	3.72E-04	8.73E-05	3.19E-04
P6	1.36E-04	1.60E-04	2.15E-04	8.91E-05	1.54E-04	2.49E-04	1.40E-04	1.49E-04
P8	8.70E-05	8.37E-05	9.10E-05	8.64E-05	8.98E-05	1.34E-04	1.98E-04	8.79E-05

*^1^Three virus lineages were set up for single PepMV-EU or mixed PepMV-EU + CH2 infections (Figure 5A; Agüero et al., 2018).*

*^2^Nucleotide diversity was estimated for each lineage and passage or pooling together (L1 + L2 + L3) all the sequences for each passage.*

*^3^A region of 430 nucleotides within the PepMV-EU RdRp coding sequence was RT-PCR amplified and deep sequenced for passages P1, P2, P4, P6, and P8.*

**TABLE 3:** PepMV-CH2 nucleotide diversity (π) along passages.

	**Single infection**				**Mixed infection**			
	**L1^1^**	**L2**	**L3**	**L1 + L2 + L3^2^**	**L1**	**L2**	**L3**	**L1 + L2 + L3**
P1^3^	1.21E-03	1.85E-03	1.07E-03	1.24E-03	3.26E-04	6.14E-04	2.82E-04	3.88E-04
P2	5.76E-04	1.99E-03	1.38E-03	1.00E-03	1.22E-03	1.19E-03	1.05E-03	1.66E-03
P4	2.28E-04	5.48E-04	2.90E-04	1.99E-04	2.96E-04	3.33E-04	1.56E-03	1.84E-03
P6	1.37E-03	1.19E-04	1.07E-03	8.97E-04	1.74E-04	2.26E-04	1.17E-03	2.09E-03
P8	1.10E-03	9.47E-04	4.78E-04	6.89E-04	3.13E-04	5.19E-04	8.71E-04	1.63E-03

*^1^Three virus lineages were set up for single PepMV-CH2 or mixed PepMV-EU + CH2 infections (Figure 5A; Agüero et al., 2018).*

*^2^Nucleotide diversity was estimated for each lineage and passage or pooling together (L1 + L2 + L3) all the sequences for each passage.*

*^3^A region of 430 nucleotides within the PepMV-CH2 RdRp coding sequence was RT-PCR amplified and deep sequenced for passages P1, P2, P4, P6, and P8.*

**TABLE 4:** Allele found at position 1312 of the PepMV-CH2 RdRp coding sequence.

		**Passage 1**	**Passage 2**	**Passage 4**	**Passage 6**	**Passage 8**
Single infections	L1	T (Asp)^2^	G (Glu); F^1^ = 0.484	T (Asp) ^2^	G (Glu); F^1^ = 0.995	G (Glu); F^1^ = 0.996
			A (Glu); F^1^ = 0.023			
	L2	T (Asp) ^2^	G (Glu); F^1^ = 0.656	G (Glu); F^1^ = 0.997	G (Glu); F^1^ = 0.996	T (Asp) ^2^
	L3	T (Asp) ^2^	G (Glu); F^1^ = 0.221	G (Glu); F^1^ = 0.993	T (Asp)^2^	G (Glu); F^1^ = 0.997
Mixed infections	L1	T (Asp) ^2^	G (Glu); F^1^ = 0.23	G (Glu); F^1^ = 0.998	G (Glu); F^1^ = 0.990	T (Asp) ^2^
	L2	T (Asp) ^2^	G (Glu); F^1^ = 0.720	G (Glu); F^1^ = 0.997	G (Glu); F^1^ = 0.995	G (Glu); F^1^ = 0.998
	L3	T (Asp) ^2^	A (Glu); F^1^ = 0.057	A (Glu); F^1^ = 0.972	A (Glu); F^1^ = 0.984	A (Glu); F^1^ = 0.986

*^1^Frequency of each nucleotide variant and amino acid encoded for each lineage and passage. When the frequency is not displayed, it means that it is higher than 0.99 for the allele in the ancestral sequence and no mutations in a frequency higher than 0.01 were found.*

*^2^Allele and amino acid in the ancestral sequence.*

## Discussion

With this work, we have uncovered a new layer of complexity in the interaction between PepMV-EU and -CH2. An asymmetric antagonism was described before (Gómez et al., 2009) and corroborated later in several reports (Agüero et al., 2018; Alcaide et al., 2020a, 2021) for simultaneous mixed infections; this interaction, on its own, can contribute to maintenance of persistent PepMV-EU and -CH2 mixed infections in tomato crops. But in nature, apart from simultaneous mixed infections, over-infections may also take place. Given the overall nucleotide similarity between the PepMV-EU and -CH2 genomes (over 80%), we expected reciprocal exclusion between isolates of the two strains. Indeed, here we showed that over-infections with PepMV-CH2 resulted in exclusion, but this was not reciprocal, as over-infections with PepMV-EU resulted in the opposite effect, increasing the accumulation of PepMV-EU as compared to single or simultaneous mixed infections. From the point of view of understanding the underlying molecular mechanisms, our observations represent a challenge. Whereas mechanisms resulting in asymmetric antagonism or asymmetric exclusion against PepMV-CH2 may overlap, the non-reciprocal synergism favoring PepMV-EU after PepMV-CH2 over-infections must be controlled differently. These data suggest that PepMV may be an interesting experimental model to study a variety of interactions among viral strains, including super-infection exclusion and synergism. From a perspective of PepMV control through the use of cross-protection, our findings come as a warning, as pre-inoculations with just one mild isolate may not confer full protection; in this regard, it is probably safer to pre-inoculate with a mixture of isolates of the two strains, as proposed by Agüero et al. (2018). Finally, from an ecological point of view, this phenomenon contributes to understanding the persistence of PepMV-EU in mixed infections in tomato crops; if initial infection foci are sufficient, asymmetric antagonism after simultaneous mixed infections, asymmetric exclusion, and non-reciprocal synergism after over-infections may feed the abundance of PepMV-EU as to persist in mixed infections in spite of its inferior *in planta* fitness in single infections (Gómez et al., 2009; Alcaide et al., 2020a). In fact, our results on the impact of mixed infections on PepMV transmission reinforce the hypothesis above. In this regard, our main purpose with the transmission experiments was to verify if mixed infections could result in transmission frequencies that deviate from expectations. Transmission models have been described over the years assuming that transmission depends primarily on virus accumulation in the inoculum source (Sacristán et al., 2011); however, most of the experimental studies in this regard have been developed with vector-borne viruses (Escriu et al., 2000; Domingo-Calap et al., 2020). The study on tobacco mosaic virus (TMV) contact transmission showed that the efficiency of transmission was mostly dependent on the age of the source leaf and on the manner in which it was infected, and no correlation was found between transmission and viral accumulation in the source inoculum (Sacristán et al., 2011). Here we used plant extracts with a broad range of concentrations of the two viruses as inoculum for mechanical transmissions, showing that indeed the titer of the virus in the inoculum positively correlates with transmission efficiency, finding a threshold of PepMV-CH2 accumulation in which the transmission may not occur. It would be interesting to use a broader range of viral accumulations in order to find whether there is also a threshold of PepMV-EU accumulation in which the transmission does not occur. Therefore, *in planta* interactions between isolates of the different strains appear to modulate the spread of the viruses through modulation of their accumulation in individual hosts. Then, the question that arises is why does PepMV-EU persist in some geographical areas and not in other areas, as described, for instance, for Mazarrón *vs* Águilas in South-eastern Spain (Alcaide et al., 2020a). Perhaps the key aspect here is provision of initial infection foci season after season; indeed, our own observations suggest that tomato cultivation in Mazarrón is more intensive than in Águilas, favoring the overlap of tomato crops all year round, thus maintaining sufficient inoculum sources constantly. But these are aspects that remain to be elucidated.

In this work, we also sought to analyze the impact of mixed infections on PepMV genetic diversification. We have addressed this aspect before by using different approaches, including comparing the PepMV-CH2 natural diversity in areas with only single infections *vs* areas with mixed infections (Alcaide et al., 2020a), and in a long-term *in planta* PepMV evolution experiment including temperature in its design as an environmental variable (Alcaide et al., 2021). Regarding the first approach, we observed more variability for PepMV-CH2 in Mazarrón (mixed infections) than in Águilas (single infections), but further experiments failed to identify selective forces explaining this observation; moreover, in a different set of analyses, we identified important migration fluxes among geographical areas (Gómez-Aix et al., 2019a), and the effect of migration may blur conclusions (Lapidot et al., 2014). Regarding the second study mentioned above, we observed complex interactions among variables including viral strain (CH2 *vs* EU), type of infection (single *vs* mixed), and temperature (Alcaide et al., 2021). After these and the above observations, we wanted to take into consideration transmission as a factor that modulates PepMV variability. For this, we took advantage of a passaging experiment that was previously conducted to check the stability of PepMV isolates in single *vs* mixed infections. In this experiment, we only analyzed the variability of the viral progeny after the final passage, and the method of choice was Sanger sequencing of a limited number of cDNA clones (Agüero et al., 2018). To obtain a deeper insight into the PepMV variability along passages, here we used samples from different passages and deep sequenced an RT-PCR amplicon. The amplicon was designed in a region where PepMV-CH2 and PepMV-EU differed the most (Gómez-Aix et al., 2019b), which was also a hypervariable region for both PepMV strains (Alcaide et al., 2021). Overall, we did not observe diversification among lineages with passaging as could be expected if genetic bottlenecks took place during transmission (Sacristán et al., 2011; da Silva et al., 2020). It is likely that the amount of virus in the inoculum was sufficient to recreate the entire swarm of virus variants passage after passage, even for mixed infections. By looking at specific mutations in the genome fragment sequenced, we observed that mutation T1312(G/A) appeared to be the only one fixed after passaging (Figure 6B). It encodes the conservative amino acid change E409D, which localizes between the methyltransferase and helicase domains of the PepMV RdRp (Aguilar et al., 2002). Amino acids E and D have similar properties, thus it is possible that mutation T1312(G/A) had little impact on the protein functionality. However, the rapid change of nucleotide frequencies in both directions, T1312G between passages 1 and 2 for all lineages, and G1312T between passage 6 and 8 for lineage L2 (Table 4), suggests an adaptive value for this mutation; this is a possibility that remains to be investigated. On the other hand, our genetic diversification analyses unveiled some clear trends; PepMV-CH2 seemed to maintain a higher variability than PepMV-EU, in agreement with previous studies by our group (Alcaide et al., 2020a, 2021); and mixed infections had an impact on the PepMV-CH2 variability, although in this case to lower it in contrast with a previous study (Alcaide et al., 2021).

## Data Availability Statement

The datasets presented in this study can be found in online repositories. The names of the repository/repositories and accession number(s) can be found below: https://www.ncbi.nlm.nih.gov/, SAMN18559886–SAMN18559945.

## Author Contributions

MA and CA conceived and designed the research and wrote the manuscript. CA performed the experiments and analyzed the data. Both authors contributed to the article and approved the submitted version.

## Conflict of Interest

The authors declare that the research was conducted in the absence of any commercial or financial relationships that could be construed as a potential conflict of interest.
