# Supplementary material for: Determinants of Persistent Patterns of Pepino Mosaic Virus Mixed Infections
Source: Front Microbiol. 2021 Jul 6;12:694492. doi: 10.3389/fmicb.2021.694492 (PMC8290496; doi:10.3389/fmicb.2021.694492)
Supplement: Supplementary file 1 [file Data_Sheet_1.docx]

Supplementary Material

##
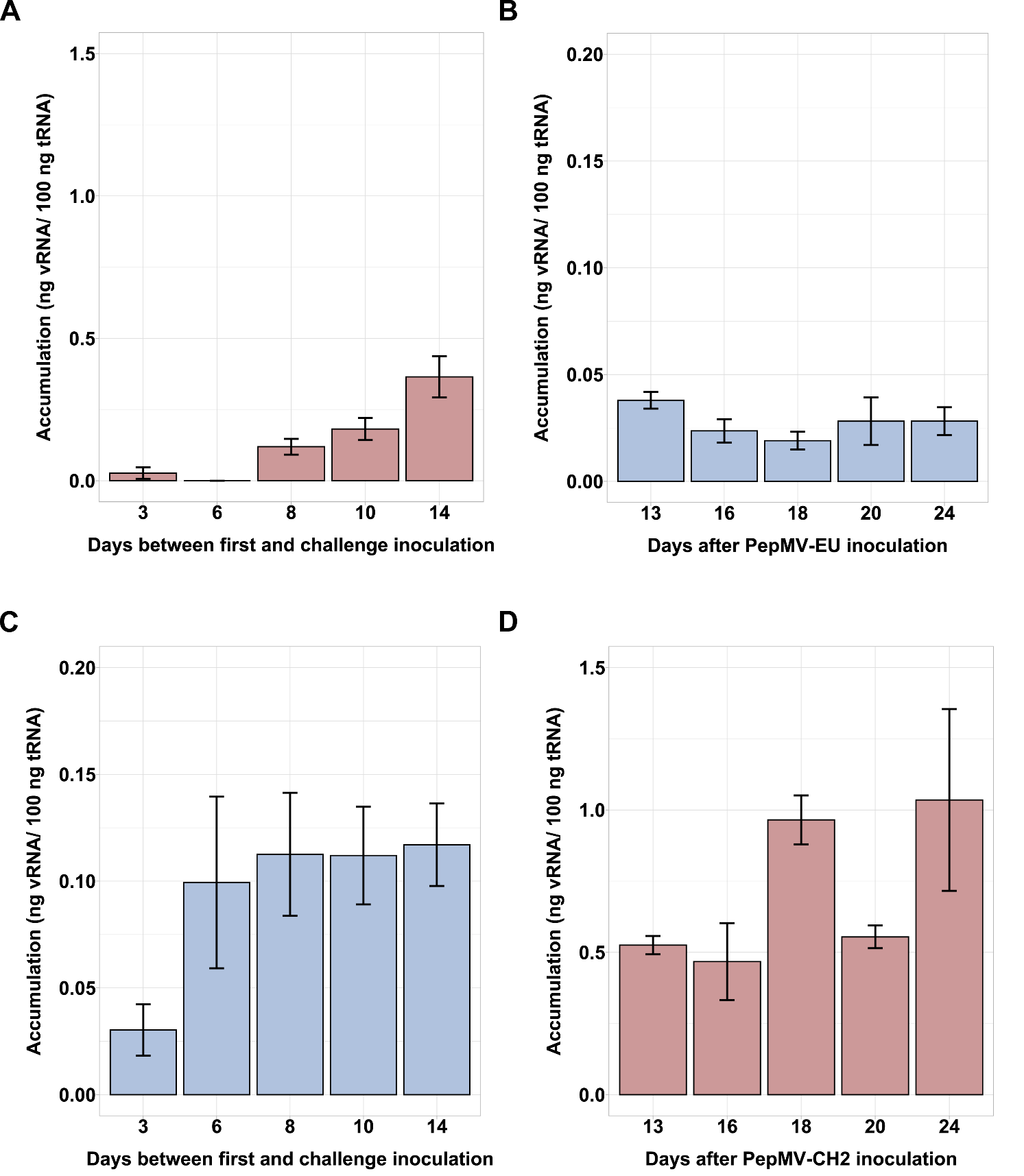
Supplementary Figures

***

***

**Supplementary Figure 1.** Effect of PepMV-CH2 **(A, D)** or PepMV-EU **(B, C)** over-infections on virus accumulation. PepMV-CH2 **(A)** and PepMV-EU **(D)** accumulation was measured in plants pre-inoculated with PepMV-EU and challenged with PepMV-CH2. Also PepMV-CH2 **(B)** and PepMV-EU **(C)** accumulation was measured in plants pre-inoculated with PepMV-CH2 and challenged with PepMV-EU. Over-inoculations were carried out at different times after pre-inoculation (3, 6, 8, 10 and 14 days). Viral accumulation was determined for 3 plants for each time point by absolute RT-qPCR and shown as ng viral RNA / 100 ng total RNA; each bar represents the mean and its standard deviation. Asterisks shows significance level (*** = p < 0.001).


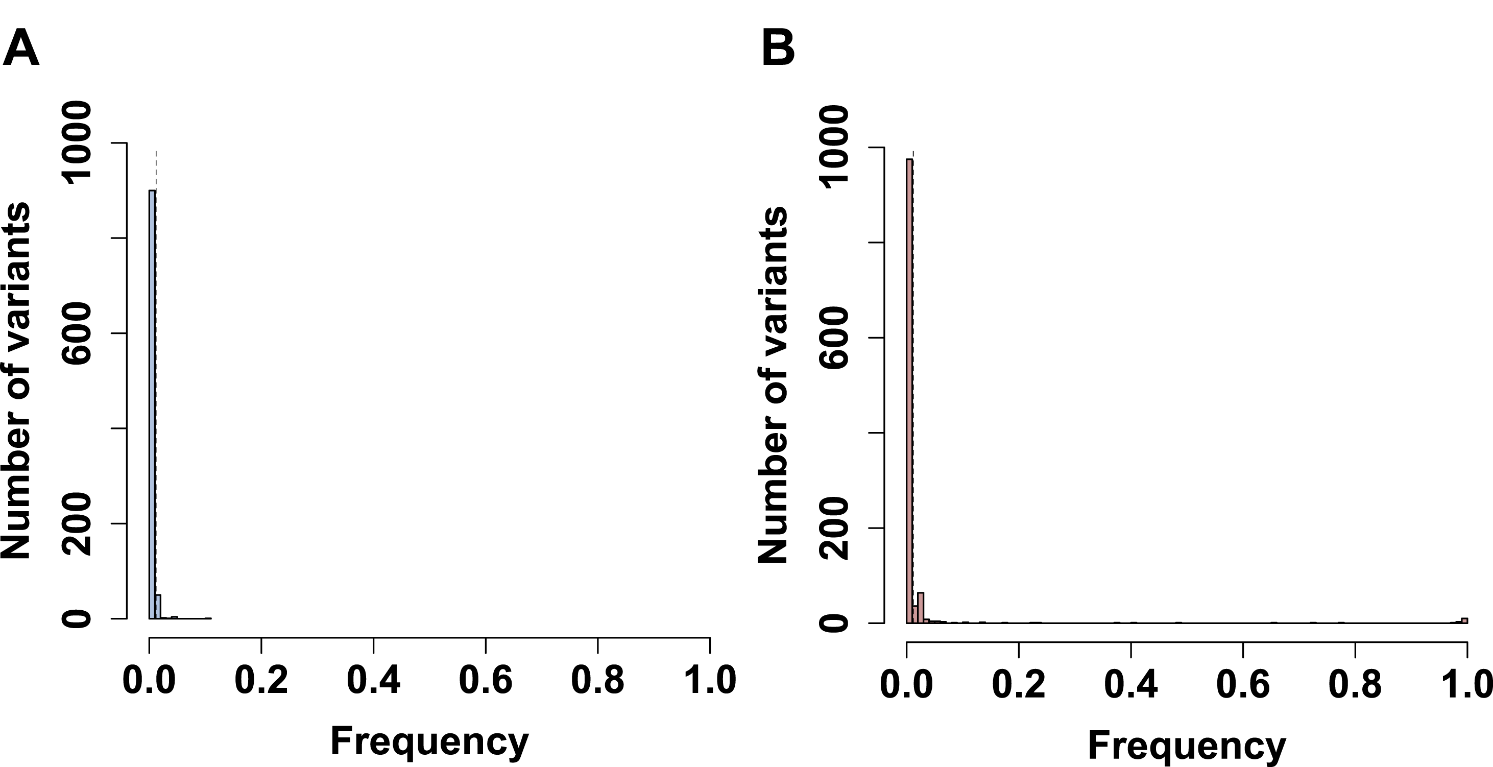
**Supplementary Figure 2.** Number of variants and their frequencies found in PepMV-EU **(A)** and PepMV-CH2 **(B)** populations. SNPs with a frequency lower than 0.01 were excluded from the analysis (left to dashed line).

**Suplementary Table 1**

| **Source plant** | **Accumulation^1^** | **Nº of infected plants** | **Strain** | **Infection type** |
| --- | --- | --- | --- | --- |
| Single EU | 1,12E-01 | 14 | EU | Single |
| Single EU dilution 1:5 | 2,23E-02 | 11 | EU | Single |
| Single EU dilution 1:25 | 4,46E-03 | 9 | EU | Single |
| Simultaneous EU + CH2 | 1,35E-01 | 14 | EU | Mixed |
| Simultaneous EU + CH2 dilution 1:5 | 2,70E-02 | 11 | EU | Mixed |
| Simultaneous EU + CH2 dilution 1:25 | 5,41E-03 | 7 | EU | Mixed |
| Overinfection CH2 | 4,70E-02 | 15 | EU | Mixed |
| Overinfection CH2 dilution 1:5 | 9,39E-03 | 14 | EU | Mixed |
| Overinfection CH2 dilution 1:25 | 1,88E-03 | 6 | EU | Mixed |
| Overinfection EU | 6,55E-01 | 18 | EU | Mixed |
| Overinfection EU dilution 1:5 | 1,31E-01 | 17 | EU | Mixed |
| Overinfection EU dilution 1:25 | 2,62E-02 | 16 | EU | Mixed |
| Single CH2 | 1,22E+00 | 16 | CH2 | Single |
| Single CH2 dilution 1:5 | 2,45E-01 | 13 | CH2 | Single |
| Single CH2 dilution 1:25 | 4,90E-02 | 12 | CH2 | Single |
| Simultaneous EU + CH2 | 7,86E-02 | 17 | CH2 | Mixed |
| Simultaneous EU + CH2 dilution 1:5 | 1,57E-02 | 17 | CH2 | Mixed |
| Simultaneous EU + CH2 dilution 1:25 | 3,14E-03 | 10 | CH2 | Mixed |
| Overinfection CH2 | 2,75E-05 | 0 | CH2 | Mixed |
| Overinfection CH2 dilution 1:5 | 5,50E-06 | 0 | CH2 | Mixed |
| Overinfection CH2 dilution 1:25 | 1,10E-06 | 0 | CH2 | Mixed |
| Overinfection EU | 1,86E+00 | 18 | CH2 | Mixed |
| Overinfection EU dilution 1:5 | 3,72E-01 | 17 | CH2 | Mixed |
| Overinfection EU dilution 1:25 | 7,43E-02 | 15 | CH2 | Mixed |

**^1^** Viral accumulation (ng viral RNA / 100 ng total RNA) in the different inoculum sources is shown, including that for two single infections, one simultaneous mixed-infection, two over-infections and their dilutions.
